# The effect of metal pollution on the life history and insecticide resistance phenotype of the major malaria vector *Anopheles arabiensis* (Diptera: Culicidae)

**DOI:** 10.1371/journal.pone.0192551

**Published:** 2018-02-06

**Authors:** Shüné V. Oliver, Basil D. Brooke

**Affiliations:** 1 Centre for Emerging Zoonotic and Parasitic Diseases, National Institute for Communicable Diseases, Johannesburg, South Africa; 2 Wits Research Institute for Malaria, School of Pathology, Faculty of Health Sciences, University of the Witwatersrand, Johannesburg, South Africa; University of Crete, GREECE

## Abstract

Metal exposure is one of the commonest anthropogenic pollutants mosquito larvae are exposed to, both in agricultural and urban settings. As members of the *Anopheles gambiae* complex, which contains several major malaria vector species including *An*. *arabiensis*, are increasingly adapting to polluted environments, this study examined the effects of larval metal exposure on various life history traits of epidemiological importance. Two laboratory strains of *An*. *arabiensis*, SENN (insecticide susceptible) and SENN DDT (insecticide resistant), were reared in maximum acceptable toxicity concentrations, (MATC—the highest legally accepted concentration) of cadmium chloride, lead nitrate and copper nitrate. Following these exposures, time to pupation, adult size and longevity were determined. Larvae reared in double the MATC were assessed for changes in malathion and deltamethrin tolerance, measured by lethal time bottle bioassay, as well as changes in detoxification enzyme activity. As defence against oxidative stress has previously been demonstrated to affect the expression of insecticide resistance, catalase, glutathione peroxidase and superoxide dismutase activity was assessed. The relative metal toxicity to metal naïve larvae was also assessed. SENN DDT larvae were more tolerant of metal pollution than SENN larvae. Pupation in SENN larvae was significantly reduced by metal exposure, while adult longevity was not affected. SENN DDT showed decreased adult size after larval metal exposure. Adult insecticide tolerance was increased after larval metal exposure, and this effect appeared to be mediated by increased β-esterase, cytochrome P450 and superoxide dismutase activity. These data suggest an enzyme-mediated positive link between tolerance to metal pollutants and insecticide resistance in adult mosquitoes. Furthermore, exposure of larvae to metal pollutants may have operational consequences under an insecticide-based vector control scenario by increasing the expression of insecticide resistance in adults.

## Introduction

Human activity has led to a large-scale increase in various environmental pollutants. Pollution in rural areas accrues primarily from agrochemicals such as fertilizer and pesticides, but also from domestic refuse, sewage and livestock excrement [1]. Urban pollutants arise from domestic waste, traffic and industry [2]. In South Africa, like many other African countries, vital economic activities such as artisanal gold mining and coal power stations are a major source of pollution [3]. Pollution in rural and urban environments typically leads to an increase in heavy metal contamination.

Mining is a source of urban metal pollution that has the potential to cause widespread contamination by runoff and rainwater seepage into water systems [4]. Increased ground transportation also results in increased levels of water soluble heavy metals (reviewed in [5]). Although rural areas are not commonly associated with metal pollution, the use of phosphate fertilizers can contribute to the increase of hazardous heavy metal trace elements [6]. In general, metal pollution is highly pervasive wherever human activity occurs. Importantly, these heavy metal contaminants have a tendency to pollute water sources [4] and this has numerous environmental and public health consequences.

Members of the *Anopheles gambiae* species complex typically prefer to breed in open, sunlit temporary bodies of water that are primarily unpolluted [7]. Therefore, reports of members of this complex breeding in polluted water represent a significant biological shift in this species [8–10]. Furthermore, this shift is not only reported in *An*. *gambiae sensu stricto*, but other members of this species complex including *An*. *arabiensis* [11, 12]. As non-pesticidal residues have been demonstrated to modulate detoxification enzyme capacity [13–15], as well as insecticide resistance phenotypes [16], this adaption could inadvertently affect biological attributes of epidemiological importance, such as insecticide susceptibility.

The importance of environmental pollution on pyrethroid resistance has been reviewed [2], and metal pollution has been named as one of the most important effectors of resistance phenotype in urban settings. The effect of metal pollution has been examined in *An*. *gambiae* and *Aedes aegypti*. Adapting to the presence of environmental metal pollutants incurs a fitness cost to the larvae. Heavy metals arrest egg hatching and compromise the integrity of the larval peritrophic matrix in *Ae*. *aegypti* larvae when naïve populations are exposed [17]. It is possible to select for metal tolerance in *An*. *gambiae* relatively rapidly under laboratory conditions [10]. However, this adaption incurs a significant biological cost, including reduced egg viability, immature survivorship and emergence as well as reduced reproductive capacity [18].

At the molecular level, the development of tolerance to metal pollutants involves protein induction shifts [10]. For example, the stress response genes coding glutathione S-transferase [19], metallothionein [20] as well as mucin and α-tubulin have been associated with metal tolerance [17, 21, 22]. Several cytochrome P450 genes, particularly in the CYP6 class, have also been associated with metal tolerance. The induction of these genes is sex-specific and although the relevant genes are expressed at their lowest levels in females, the gene induction was female specific, with males incurring a significant reduction in the expression of CYP6M2, CYP6P3 and CYP6Z1 [23].

Although adaptation to metal pollution has also been reported in *An*. *arabiensis* [11, 12], very little work has been done on this species’ molecular response to pollution [2]. *Anopheles arabiensis* is a dominant malaria vector species in southern Africa, including South Africa [24]. The variable feeding and resting behaviours of this species presents a challenge to vector control methods that are based on indoor applications of insecticide [25, 26]. This is because outdoor biting and resting can lead to sustained low-level residual transmission in a control setting [27], which threatens South Africa’s malaria elimination agenda.

This aim of this study was to understand the effects of heavy metal pollution on various life history traits as well as the expression of insecticide resistance in *An*. *arabiensis*.

## Methods

### Materials

All experiments were performed in the Botha de Meillon insectary, National Institute for Communicable Diseases, Johannesburg. Basic *Anopheles* colony rearing and feeding conditions were as described in [28]. The baseline strain used in this study was SENN, which was colonised from Sennar, Sudan, in 1980. This strain is mostly insecticide susceptible, with low-level permethrin resistance [29]. The SENN DDT strain was selected from the SENN strain and has been continuously exposed to DDT since 1995. It currently displays resistance to DDT, permethrin, deltamethrin, λ-cyhalothrin and malathion [29, 30]. Resistance in this strain is due to elevated detoxification enzymes as well as the L1014F mutation [31, 32] Elevated oxidative stress enzymes have also been implicated in the construction of the resistant phenotype [33].

### Comparative metal-induced lethality in insecticide resistant and susceptible *An*. *arabiensis*

Three representative metals were used in this study, based on previous research [10]. Ten percent stock solutions of cadmium chloride, copper nitrate and lead nitrate were prepared. The minimum lethal concentrations of these metal salts were determined by using standard WHO larviciding protocols [34]. As lead was significantly less toxic, a slight variation in concentration range was used to determine the lethal dose of lead nitrate, with working concentrations of 100–16000 ppm. The specific concentrations used for these experiments are given as supplementary materials. Twenty-five fourth instar larvae were used per concentration, and 4 replicates were performed as per WHO recommendations. Lethal concentrations inducing 50% mortality (LC_50_) were determined by Probit analysis [35], using IBM SPSS v21(IBM Corp. Released 2012. IBM SPSS Statistics for Windows, Version 21.0. Armonk, NY: IBM Corp.).

### The effect of metal pollution on larval development

The development of SENN and SENN DDT larvae when exposed to metal pollutants was assessed. Newly emerged larvae, less than 24 hours old, were exposed to the maximum acceptable toxicant concentration (MATC) of the relevant metal salts. Larvae reared in untreated water served as a control. MATCs for the representative metal salts were defined in [22] (0.36μg/L for cadmium chloride, 1.86μg/L for copper nitrate and 4.39μg/L for lead nitrate). All larvae were fed the same amount of food. Larvae were monitored until pupation, and the percentage of pupation on the day of maximal pupation (the day the most pupae emerged; in this study—day 9) was determined. This study was replicated three times from three different egg batches, resulting in a total of 9 replicates, with a concurrent control (rearing in untreated water) with each replicate.

### The effect of larval metal pollution on adult longevity

To assess the effect of larval metal pollution on subsequent adult longevity, 100 24-hour-old SENN and SENN DDT 1^st^ instar larvae were exposed to the MATC of cadmium chloride, copper nitrate or lead nitrate, with corresponding larvae in clean water serving as a control. The larvae were fed the same amount of food as described in [31]. Adults that were obtained from these treatments were used for longevity assays. Thirty males and 30 females were assessed for longevity. Cadavers were removed daily, and the adults were allowed *ad libitum* access to 10% sucrose, but never allowed a blood meal for the course of their lifetime. Survival was monitored until all individuals were dead. This experiment was replicated 3 times from 3 different cohorts. Longevity was assessed using the Kaplan-Meier estimator with the log-rank test used as a measure of significance.

### The effect of larval metal exposure on adult size

Fifty 24-hour SENN and SENN DDT 1st instar larvae were exposed to the MATC of either cadmium chloride, copper nitrate and lead nitrate or clean water (control). The larvae were fed the same amount of food until emergence as adults. The adults were harvested and cold-killed. The wings were removed and affixed to a microscope slide. Wing-length was used as a proxy for size, with the wing measured from the wing tip to the allula [36]. Wing-length was measured at 200x magnification. This experiment was replicated three times from three different cohorts, and means were compared by 1-way analysis of variance.

### The effect of larval metal exposure on adult insecticide tolerance

SENN and SENN DDT larvae were used in this experiment. As SENN is significantly more susceptible to insecticides an accurate lethal dose could not be determined. As such a lethal time approach was adopted. Due to the aforementioned difference in resistance phenotype, different timescales were employed to determine the lethal times. Two hundred and fifty 24-hour old 1st instar larvae were reared in the MATC of either cadmium chloride, copper nitrate or lead nitrate, with control larvae reared in untreated water. The larvae were fed the same amount of food until pupation. Adults were harvested at 3 days, and females were not allowed to blood feed. The adults were then subjected to CDC bottle bioassay insecticide exposures to determine lethal time to 50% mortality (LT50). SENN adult females were exposed to either 0.001% malathion or deltamethrin for 2,4, 8, 16 or 32 minutes. SENN DDT adults were exposed to 0.01% malathion or deltamethrin for 10, 20, 40 or 80 minutes. This experiment was replicated three times from three different cohorts. Differences in LT50 between adult cohorts arising from pollutant treatments were compared by 1-way ANOVA.

### The effect of larval metal exposure on adult enzyme activity

SENN and SENN DDT larvae were prepared as for the adult insecticide tolerance experiments. At 3 days old, non-blood fed adult females were cold-killed. Adults were homogenised in either PCR grade water or 0.1M Potassium phosphate pH 7.0. Haeme peroxidase, glutathione S-transferase (GST) and α- and β-esterase activity levels were assessed in order to determine detoxification enzyme activities in association with exposure to heavy metals. Superoxide dismutase, glutathione peroxidase and catalase activities were determined as representative of oxidative stress defence enzyme activity. Enzyme activity by treatment was determined as described in [37]. In brief, all assays employed a calorimetric assessment of enzyme activity. Haeme perxoxidase activity was determined by tetramethyl benzidine peroxidation, GST activity by rate of 1-Chloro 2–4 Dinitrobenzene conjugation with reduced glutathione, and esterase activity by quantification of the hydrolosis of α and β-napthyl acetate to α and β-napthol. These products were spectrophotometrically quantified at 650, 340 and 575 nm respectively. Superoxide dismutase activity was determined using a commercial kit (Sigma Aldrich: 19160) as per manufacturers’ instructions. Glutathione peroxidase activity was determined as function of Nicotinamide adenine dinucleotide phosphate (NADPH) consumption measured at340nm and catalase activity determined as a function of hydrogen peroxide consumption measured at 570nm. All calorimetric analysis was performed on performed on a Multiskan Ascent 96 well plate reader (Thermo Scientific).

## Results

### Insecticide resistant phenotype and comparative metal lethality

**Fig 1:**
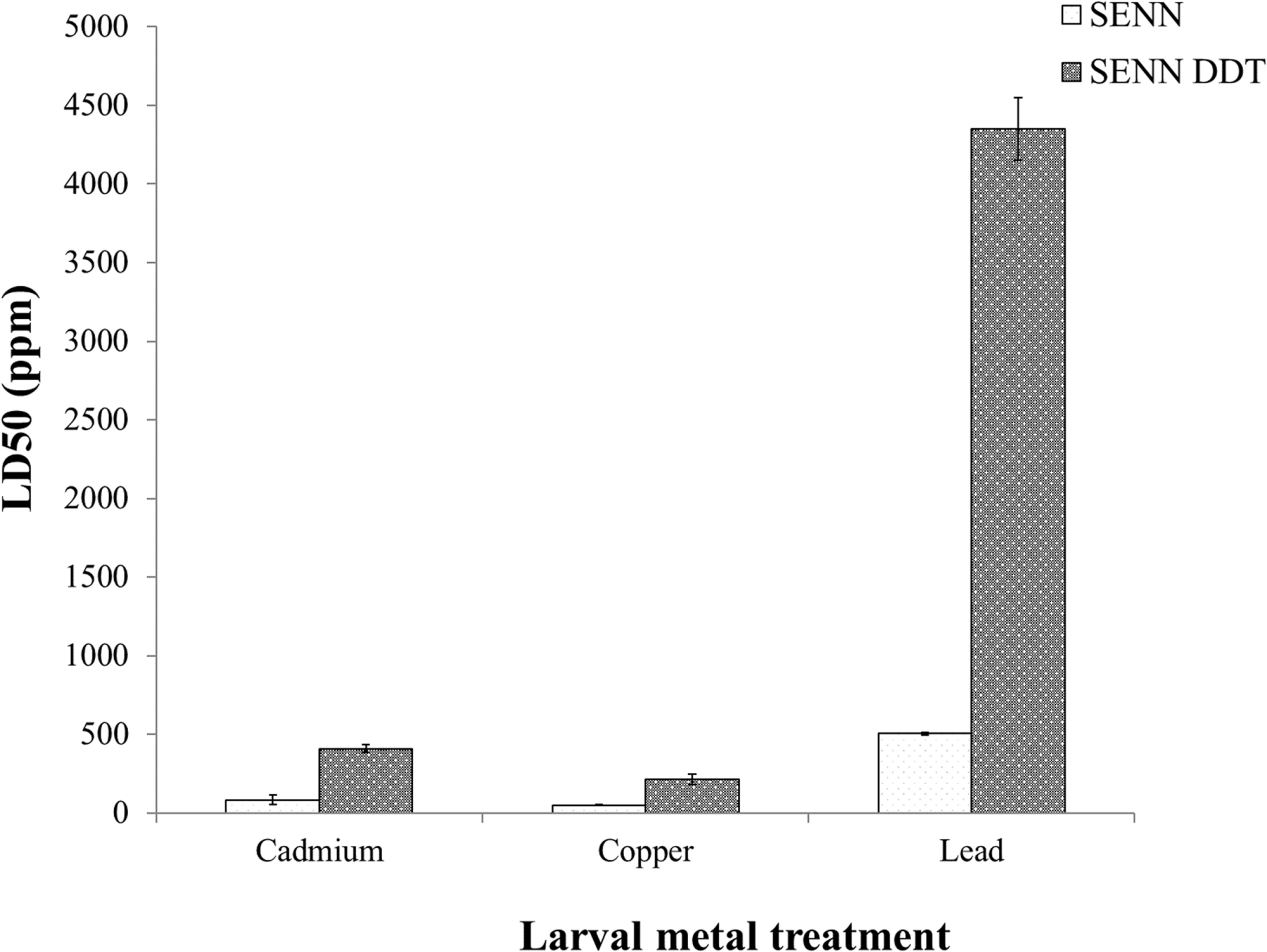
Relative toxicity of heavy metals on insecticide resistant and susceptible *Anopheles arabiensis* laboratory strains. The insecticide resistant *An*. *arabiensis* strain SENN DDT showed a significantly higher tolerance for metal exposure than the insecticide susceptible SENN strain, based on LD50s. Copper nitrate was the most toxic metal to both strains, while both strains showed a high tolerance to lead nitrate. Significant differences (p<0.05) are indicated by an asterisk (*).

SENN DDT larvae showed significantly higher tolerance to metals, as measured by differences in lethality. The respective LC50s of SENN DDT were 409.41, 212.87 and 4349.10 parts per million (ppm) for cadmium chloride, copper nitrate and lead nitrate respectively as opposed to 82.93, 50.53 and 504.36ppm respectively for SENN. SENN DDT LD50 values are significantly higher than those of SENN (1-way ANOVA: p<0.01; df = 5; F = 401) (Fig 1).

### The effect of metal pollution on larval development

**Fig 2:**
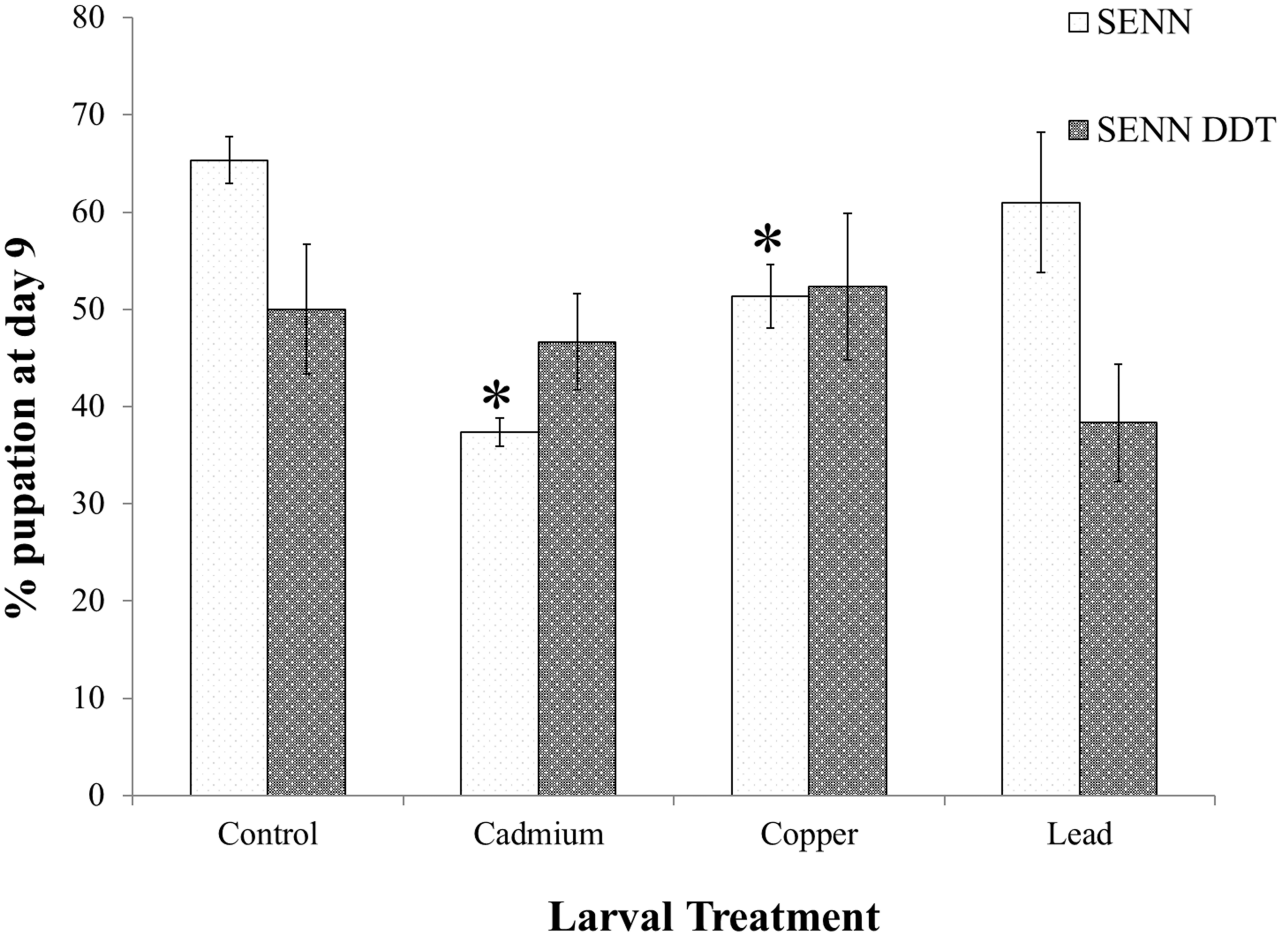
The effect of metal pollution on larval development in insecticide susceptible and resistant *Anopheles arabiensis*. SENN and SENN DDT larvae were reared in the maximum acceptable toxicity concentration (MATC) of cadmium chloride, copper nitrate and lead nitrate. Both strains had the highest number of pupae emerging on day 9, regardless of whether they were reared in metal polluted or untreated control water. There was variation in the total number that pupated on day 9. Larval cadmium chloride and copper nitrate treatment significantly reduced the percentage that pupated on day 9 in the insecticide susceptible SENN strain, but not the resistant SENN DDT strain. Lead nitrate treatment did not affect pupation in either strain.

For both strains, maximal pupation was reached on day 9, regardless of treatment. For SENN DDT, the mean percentage of larvae that pupated on day 9 did not differ between treatments; control 50%, cadmium chloride 46.7%, copper nitrate 52.3% and lead nitrate 38.3% (1-way ANOVA: p = 0.47; df = 3; F = 0.93). In contrast, while lead nitrate did not result in a significant decrease in pupation rate compared to the control (control: 65.3%; lead nitrate: 61.0%) (2-sample t-test: p = 0.62; df = 2.4; T = 0.57), copper nitrate (47.5%) and cadmium chloride (37.3%) treatments did result in a significant decrease in mean percentage pupation at day 9 (copper nitrate—2-sample t-test: p = 0.03, df = 3, T = 3.90; cadmium chloride– 2-sample t-test: p<0.01, df = 3.3, T = 9.97) (Fig 2).

### The effect of metal pollution at the larval stage on adult longevity

**Fig 3:**
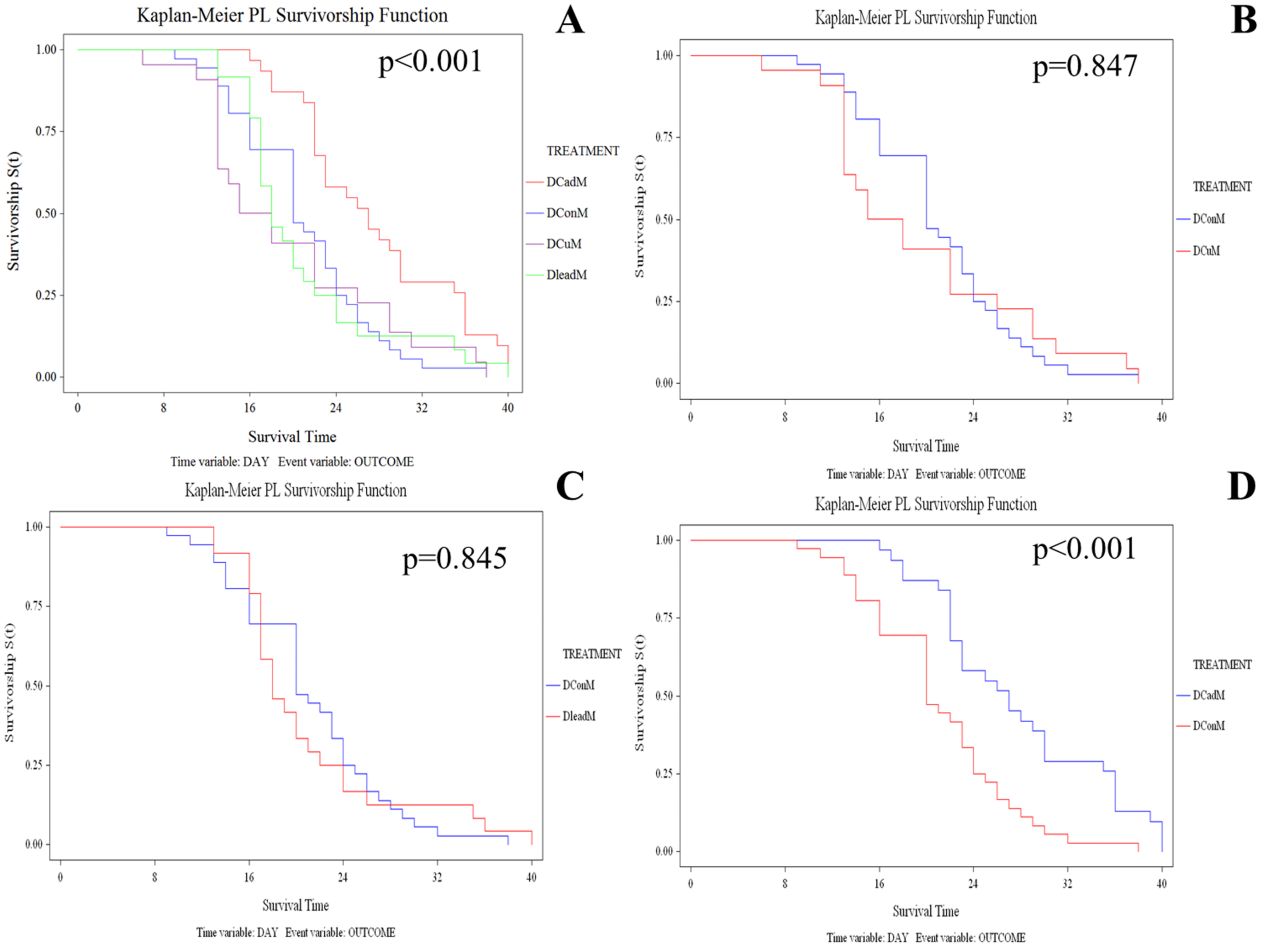
The effect of larval metal exposure on adult longevity in insecticide susceptible and resistant *Anopheles arabiensis*. Larval metal exposure did not affect adult longevity in insecticide susceptible SENN males or females, and did not affect the longevity of insecticide resistant SENN DDT females. SENN DDT males, however, did show an increase in longevity after larval metal treatment (A). That change was not due to copper nitrate (B) or lead nitrate (C) treatment, but was due to larval cadmium treatment (D).

Larval metal exposure did not have a marked effect on adult longevity. No larval metal treatments affected adult longevity in SENN males (Log-rank test: p = 0.70; χ^2^ = 1.42; df = 3) or SENN females (Log-rank test: p = 0.35; χ^2^ = 3.30; df = 3) compared to untreated controls. Similarly, SENN DDT females were not affected (Log-rank test: p = 0.51; χ^2^ = 2.31; df = 3). However, adult longevity in male SENN DDT was affected (Log-rank test: p<0.01 χ^2^ = 17.28; df = 3) (Fig 3A). When examining each treatment individually it was found that this effect was not due to larval copper treatment (Log-rank test: p = 0.85; χ^2^ = 0.04; df = 1) (Fig 3B) or lead treatment (Log-rank test: p = 0.85; χ^2^ = 0.04; df = 1) (Fig 3C). The significant change was an increase in male longevity after cadmium treatment (Log-rank test: p<0.01; χ^2^ = 11.97; df = 1) (Fig 3D).

### The effect of metal pollution at the larval stage on adult size

**Fig 4:**
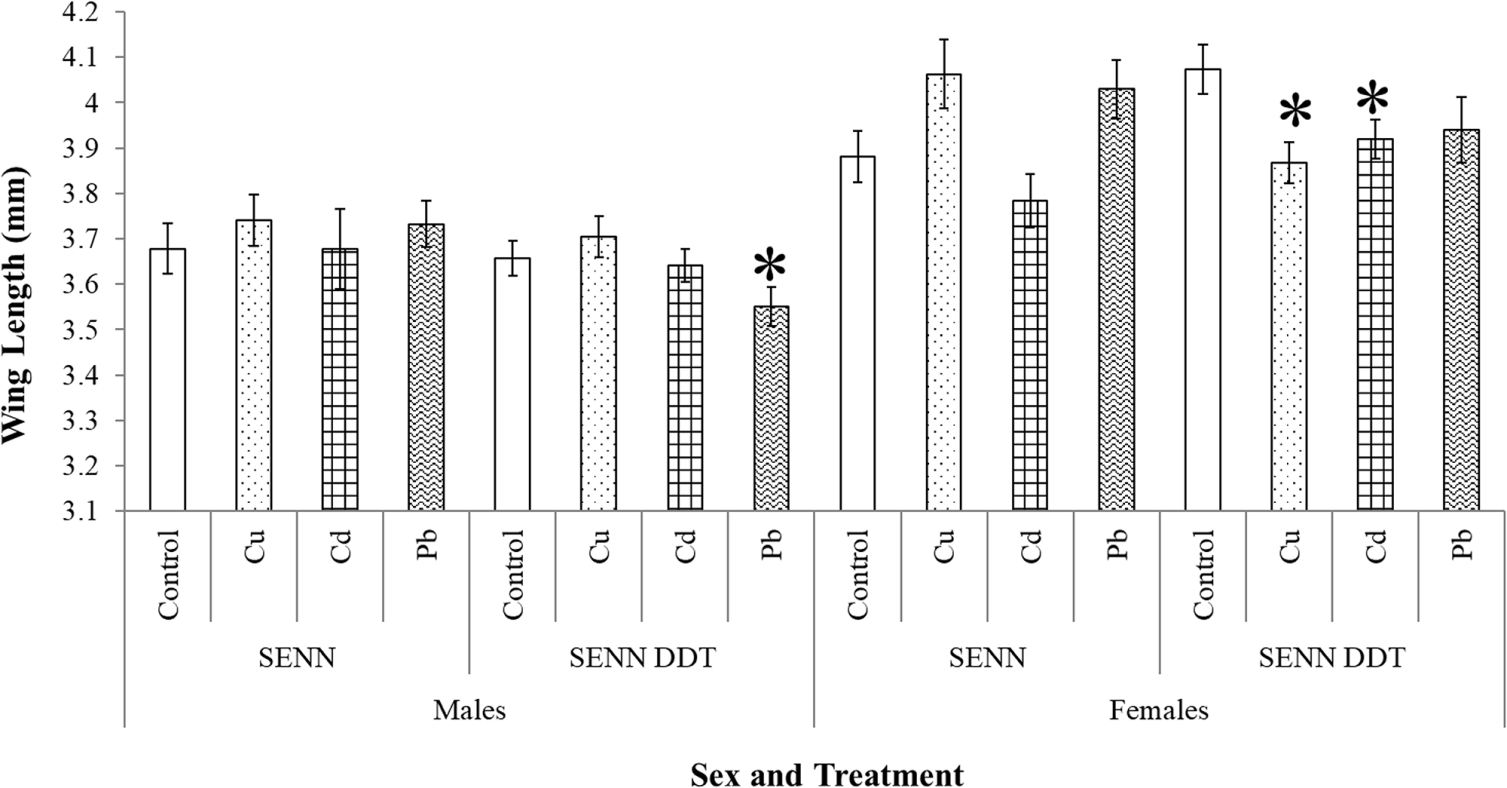
The effect of larval metal exposure on adult size in insecticide susceptible and resistant *Anopheles arabiensis*. Although larval metal treatment had no significant effect on adult size in the insecticide susceptible SENN strain, it did result in significant changes in the size of adults of the resistant SENN DDT strain. SENN DDT males that emerged from larvae exposed to lead nitrate were significantly smaller than those reared in untreated water. Similarly, SENN DDT females that eclosed from larvae that were exposed to copper nitrate and cadmium chloride were significantly smaller than adult females reared in untreated water. Significant differences from the control (p<0.05) are indicated by an asterisk (*).

Larval metal pollution had no significant effect on the size of SENN adults, regardless of gender (1-way ANOVA: males: p = 0.85, df = 3, F = 0.26; females: p = 0.03, df = 3, F = 3.59, Tukey’s post hoc test, no significant pairwise difference, critical Q = 3.87). In contrast, SENN DDT adult size was affected by larval metal pollution, although it differed by gender. SENN DDT males were not affected (1-way ANOVA: p = 0.07, df = 3, F = 2.38). Copper nitrate and cadmium chloride larval exposure significantly decreased adult size in females (1-way ANOVA: p = 0.01, df = 2, F = 4.94) (Fig 4).

### The effect of metal exposure at the larval stage on adult insecticide tolerance

**Fig 5:**
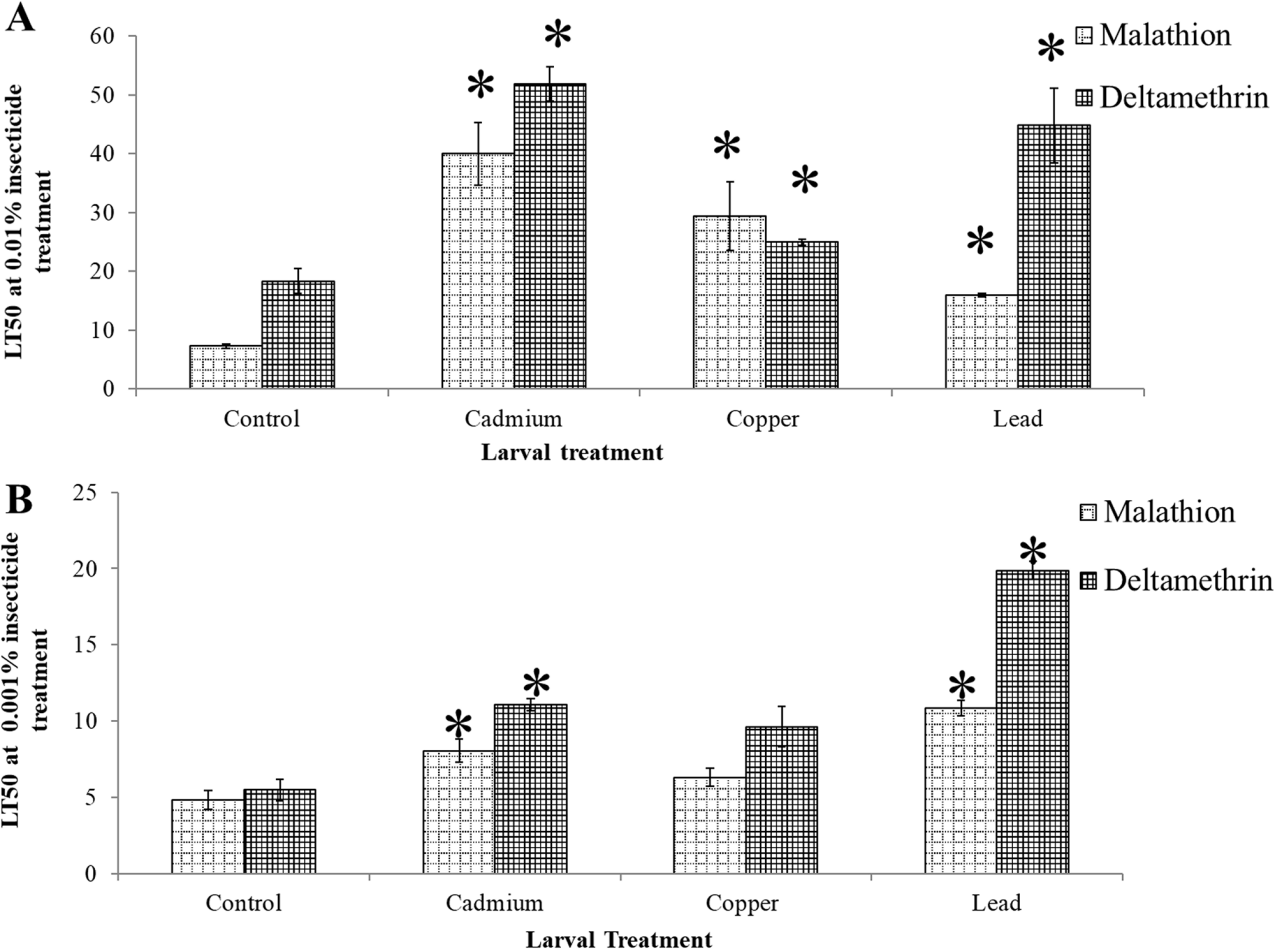
The effect of larval metal exposure on the insecticide tolerance of insecticide susceptible and resistant *Anopheles arabiensis* females. Larval metal exposure resulted in increased tolerance of malathion and deltamethrin in the insecticide resistant SENN DDT strain. All larval metal treatments resulted in a significantly increased lethal time (LT50s) in adult females (A). Larval treatment with cadmium chloride and lead nitrate resulted in a significant increase in malathion and deltamethrin tolerance of adult females of the insecticide susceptible SENN strain. Copper nitrate did not result in a change in tolerance to either insecticide in the SENN strain. Significant changes from the control (p<0.05) are indicated by asterisk (*).

Exposure to metal pollution at the larval stage had a marked effect on subsequent insecticide tolerance in adult mosquitoes. In the SENN DDT strain, exposure of larvae to all three metal treatments significantly increased adult insecticide tolerance. For malathion, lead treatment resulted in a 2.2 fold increase in LT50 (2 sample t-test: p<0.01, df = 3.8, T = -17.11), copper treatment in a 4.0 fold increase (2 sample t-test: p<0.01, df = 2.1, T = -14.87) and cadmium in a 5.5 fold increase (2 sample t-test: p<0.01, df = 4, T = -9.30). Similarly, for deltamethrin lead treatment resulted in a 2.5 fold increase in LT50 (2 sample t-test: p = 0.04, df = 3.8, T = 3.96), copper treatment in a 1.4 fold increase (2 sample t-test: p = 004, df = 5, T = -2.72) and cadmium in a 5.5 fold increase (2 sample t-test: p<0.01, df = 3.8, T = -6.10) (Fig 5A).

For the SENN strain, although exposure to copper nitrate did not result in a significant increase in adult LT50 (2-sample t-test: deltamethrin- p = 0.21, df = 2.4, T = -1.71; malathion: p = 0.13, df = 3.5, T = -1.97), cadmium chloride did result in a significant increase in LT50 (2-sample t-test: deltamethrin-p = 0.0.1, df = 5.0, T = -4.45; malathion: p = 0.02, df = 5.0, T = -3.44), as did lead nitrate (2-sample t-test: deltamethrin- p = 0.02, df = 6.0, T = -3.19; malathion: p = 0.02, df = 5.5, T = -3.49) (Fig 5B).

### The effect of larval metal treatment on adult detoxification enzyme activity

**Fig 6:**
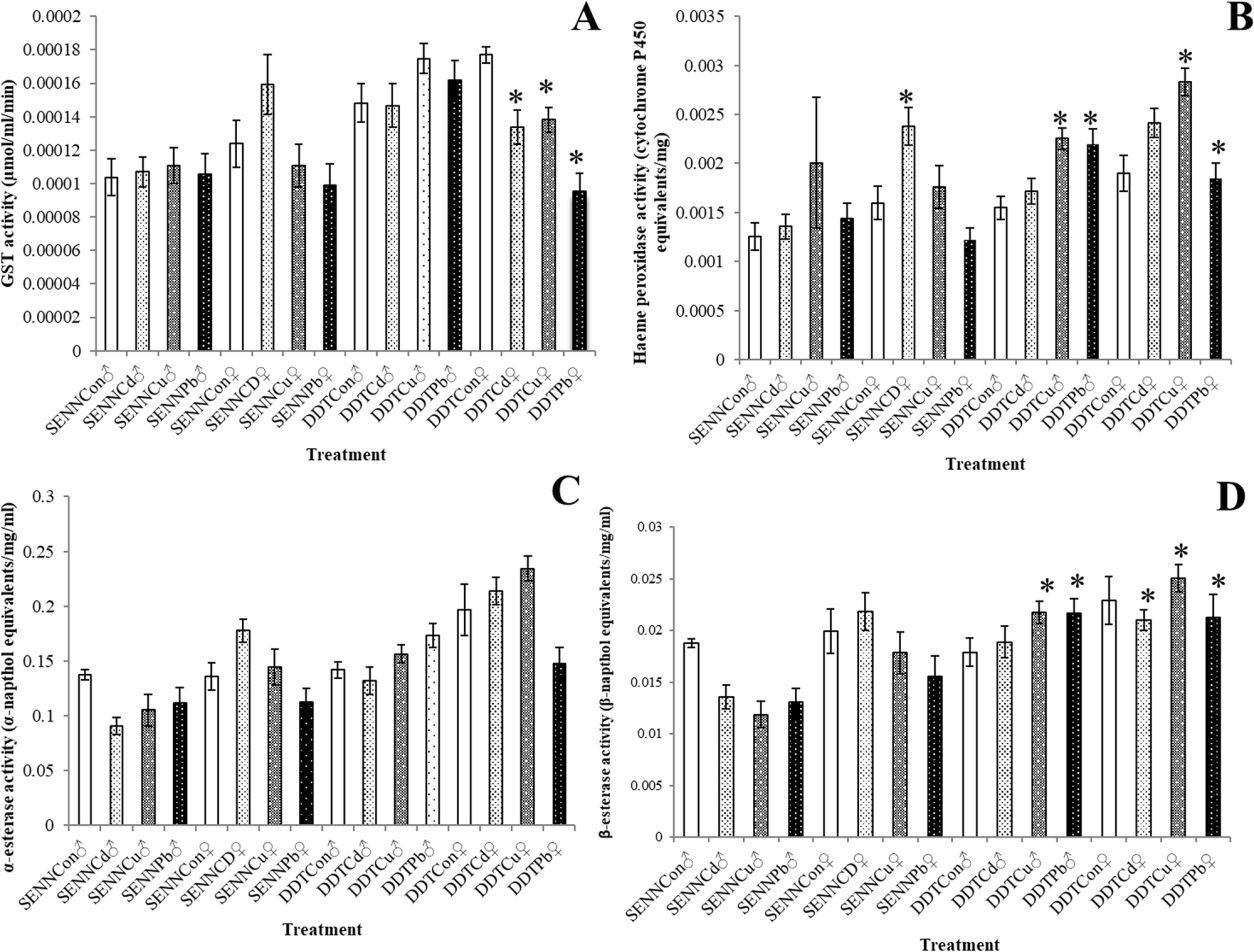
The effect of larval metal exposure on the detoxification enzyme activity of insecticide susceptible and resistant *Anopheles arabiensis* adults. Glutathione S-transferase (GST) activity was significantly decreased in SENN DDT females after all metal treatments, but no other treatments resulted in changes in GST activity (A). Cytochrome P450 activity, however, was significantly affected by certain treatments. SENN females showed a significant increase in activity after larval cadmium exposure. SENN DDT adult males showed significantly increased P450 activity after larval copper nitrate and lead nitrate exposure, while SENN DDT female enzyme activity was significantly increased after cadmium chloride and lead nitrate exposure (B). Neither α-esterase activity (C) nor β-esterase activity (D) was significantly altered in adults after larval metal exposure. Significant changes from control treatment (p<0.05) are indicated by asterisk (*).

The effect of larval metal treatment on subsequent adult detoxification enzyme activity was varied. For GST activity, larval metal exposure had no effect on SENN females (2 sample t-test: cadmium- p = 0.12, df = 43.4, T = -1.56; copper- p = 0.50, df = 45.7, T = 0.68; lead- p = 0.19, df = 45.8, T = 1.32) or males (2 sample t-test: cadmium- p = 0.82, df = 44.7, T = -0.22; copper- p = 0.66, df = 46.0, T = -0.45; lead- p = 0.90, df = 45.5, T = -0.12). This was also true for SENN DDT males (2 sample t-test: cadmium- p = 0.92, df = 45.3, T = 0.10; copper- p = 0.07, df = 43.3, T = -1.84; lead- p = 0.41, df = 45.9, T = -0.82). There was a significant effect on SENN DDT females where all larval metal treatments decreased GST activity (2 sample t-test: cadmium- p<0.01, df = 46, T = 4.46; copper- p<0.01, df = 46, T = 3.83; lead- p = 0.19, df = 46, T = 7.17) (Fig 6A).

Cytochrome P450 activity was the most affected by exposure of larvae to metals. Although SENN males showed no significant changes (1-way ANOVA: p = 0.45, df = 3, F = 0.90), cadmium treatment did result in a significant increase in P450 activity in SENN females (2 sample t-test: cadmium- p = 0.01, df = 45.3, T = -3.03; copper- p = 0.56, df = 41.6, T = -0.59; lead- p = 0.08, df = 45.8, T = 1.18). For SENN DDT, lead treatment did not induce significant changes, while cadmium and copper treatment resulted in significant increases in enzyme activity in females (2 sample t-test: cadmium- p = 0.03, df = 43.9, T = -2.19; copper- p<0.01, df = 43.1 T = -4.04; lead- p = 0.82, df = 44.6, T = 0.22). Although cadmium treatment did not increase P450 activity in females, copper and lead treatment did result in an increase in enzyme activity (2 sample t-test: cadmium- p = 0.35 df = 45.54, T = -0.94; copper- p<0.01, df = 45.9, T = -4.33; lead- p<0.01, df = 42.9, T = -3.32) (Fig 6B).

Alpha esterase activity was not affected by larval treatment with metals in either of the strains (1-way ANOVA: SENN males—p = 0.20, F = 1.56, df = 3; SENN females—p = 0.12, F = 1.99, df = 3; SENN DDT males—p = 0.14, F = 1.89, df = 3; SENN DDT females—p = 0.20, F = 1.56, df = 3). Beta esterase activity was not increased in SENN males (1-way ANOVA: p = 0.14, F = 1.82, df = 3) or SENN DDT females (1-way ANOVA: p = 0.09, F = 2.27, df = 3). In contrast, larval metal treatment did increase beta esterase activity in SENN females (1-way ANOVA: p = 0.02, F = 3.85, df = 3) and SENN DDT males (1-way ANOVA: p = 0.01, F = 4.24, df = 3).

### The effect of larval metal treatment on adult oxidative stress enzyme activity

**Fig 7:**
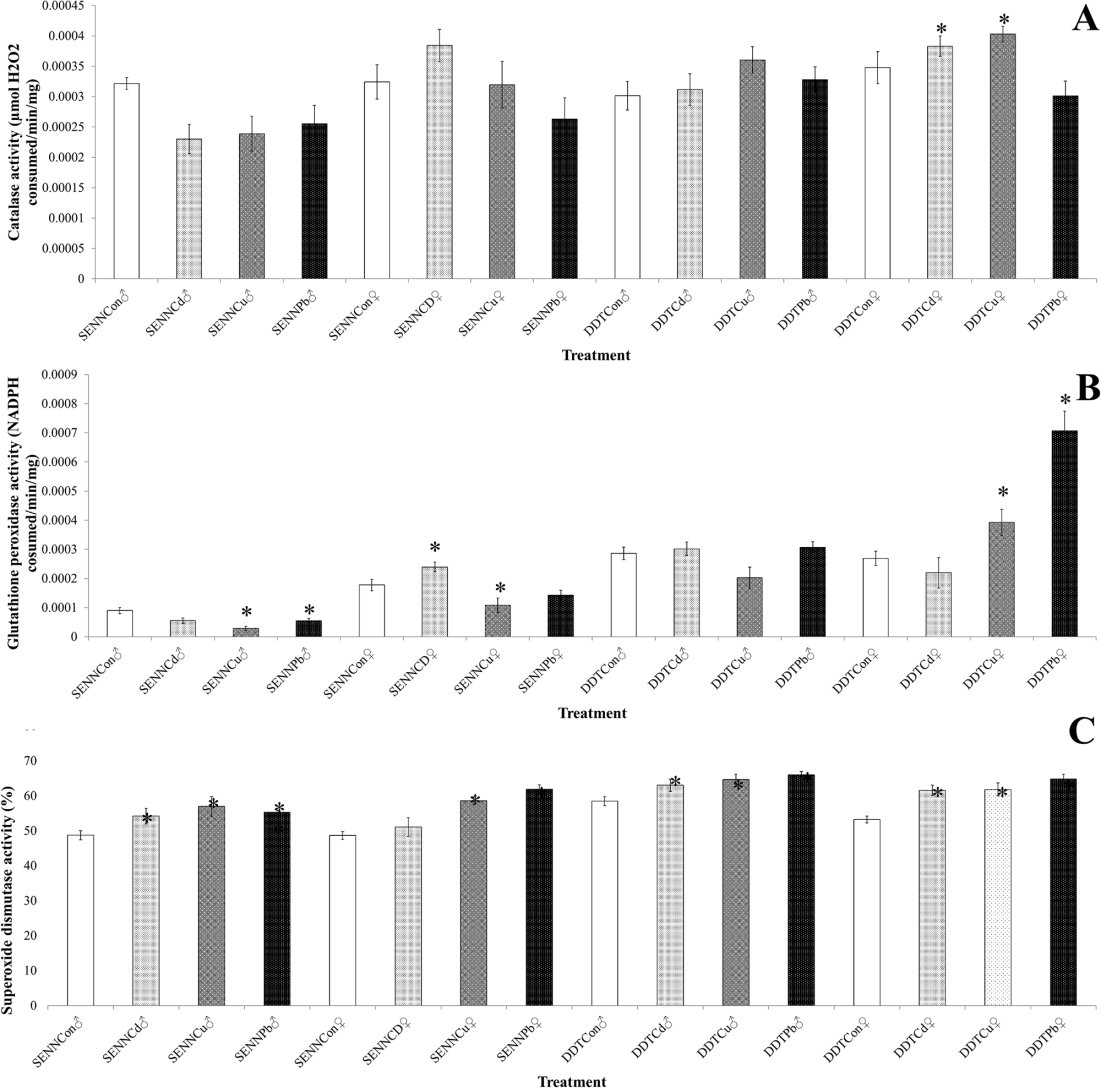
The effect of larval metal exposure on the oxidative stress enzyme activity of insecticide susceptible and resistant *Anopheles arabiensis* adults. Larval metal exposure had a highly variable effect on the three classes of oxidative stress defence enzymes. Catalase was least affected by larval metal exposure, with only SENN DDT females showing increased activity after larval copper nitrate and cadmium chloride exposure (A). Glutathione peroxidase activity showed the greatest variability after larval metal treatment. In the insecticide susceptible SENN strain, all three larval metal treatments significantly decreased peroxidase activity in adult males. In SENN females, although copper nitrate treatment significantly decreased peroxidase activity, cadmium treatment significantly increased adult peroxidase activity. In contrast, larval metal treatment had no effect on adult peroxidase activity in males of the resistant SENN DDT strain, while lead nitrate treatment resulted in a significant increase in activity in females (B). Superoxide dismutase activity was the most uniformly affected, with only lead treatment failing to elicit a significant increase in superoxide dismutase activity (C). Significant changes from the control (p<0.05) are indicated by an asterisk (*).

Larval metal exposure did not have a significant effect on the catalase activity of SENN females (1-way ANOVA: p = 0.13, df = 3, F = 1.95) or males (1-way ANOVA: p = 0.18, df = 3, F = 1.65). Similarly, SENN DDT males did not show increased catalase activity (1-way ANOVA: p = 0.31, df = 3, F = 1.21). In contrast, SENN DDT female catalase activity was strongly affected by larval cadmium and copper exposure (1-way ANOVA: p<0.01, df = 3, F = 138), but not lead exposure (1-way ANOVA: p = 0.24, df = 1, F = 1.42) (Fig 7A).

Larval metal exposure variably affected Glutathione peroxidase activity. For the SENN strain peroxidase activity was decreased in males (1-way ANOVA: p<0.01, df = 3, F = 8.61) and females treated with copper and cadmium (1-way ANOVA: p<0.01, df = 2, F = 10.0), but not lead (1-way ANOVA: p = 0.19, df = 1, F = 1.77). In contrast, peroxidase activity was varied in SENN DDT. For SENN DDT males, lead and cadmium treatment did not have a significant effect on peroxidase activity (1-way ANOVA: p = 0.78, df = 2, F = 0.25), but larval copper treatment resulted in a significant decrease (1-way ANOVA: p = 0.05, df = 1, F = 3.84). For SENN DDT females, cadmium treatment did not have a significant effect on peroxidase activity (2-sample t-test: p = 0.39, df = 46, T = 0.86), but copper treatment did result in a significant increase in activity (2-sample t-test: p = 0.02, df = 46, T = -2.41) as did lead treatment (2-sample t-test: p<0.01, df = 46, T = -6.07) (Fig 7B).

Superoxide dismutase activity was most affected by larval metal treatment. For SENN females, cadmium treatment did not have a significant effect (2-sample t-test: p = 0.39, df = 46, T = -0.86), while copper and lead treatment resulted in a significant increase in peroxidase activity (1-way ANOVA: p<0.01, df = 2, F = 35.9). For males, all three lead treatments resulted in a significant increase in enzyme activity (1-way ANOVA: p = 0.02, df = 3, F = 3.41). Similarly, all three metal treatments significantly increased peroxidase activity in both SENN DDT males (1-way ANOVA: p<0.01, df = 3, F = 12.6) as well as females (1-way ANOVA: p<0.01, df = 3, F = 6.28) (Fig 7C).

## Discussion

Metal pollution is one of the most important anthropogenic pollutants *Anopheles* mosquitoes are exposed to, both in urban and rural areas. Members of the *Anopheles gambiae* complex are known to breed in unpolluted, temporary bodies of water [7]. These include important malaria vector species that have adapted to these polluted environments [8, 11, 12] and these adaptations are likely to affect life history traits of epidemiological importance [2, 13].

The concentrations of the metals chosen in this study represent amounts that are legally tolerable tolerated in water bodies, showing that even these amounts exert a significantly measurable biological effect on vector mosquitoes i.e.in water bodies. data from this study suggest that increased pollution augments the expression of insecticide resistance in resistant populations. Although this study did not show an overall effect on adult longevity which is therefore unlikely to affect transmission dynamics [38, 39], the data do indicate that insecticide resistant individuals are advantaged under polluted conditions owing to their enhanced detoxification capabilities. This is because insecticide resistant mosquitoes have a higher capacity to cope with pollutants as demonstrated by significantly higher lethal doses of all three metals. This confirms the findings of Poupardin *et al*. [13]. Importantly, besides being able to survive higher doses of larval metal exposure, insecticide resistant mosquitoes also do not show increased costs to fitness (such as reduced adult longevity) following exposure to metal pollutants as compared to insecticide susceptible mosquitoes [40]. This dynamic suggests that metal-polluted environments may inadvertently select for or enhance resistance to insecticides in vector populations, which could affect subsequent malaria transmission intensity in insecticide-based control settings depending on the strength of the resistance phenotypes concerned.

In terms of life history traits there appears to be a size trade-off in adult insecticide resistant individuals which do not appear to be associated with decreased larval development time i.e. insecticide resistant mosquitoes that eclose from pollutant exposed larvae are generally smaller. The direct effect of this size differential on malaria transmission is unclear because there are hypotheses that suggest that smaller adults blood feed more frequently (so as to augment their energy reserves and complete their gonotrophic cycles) causing a higher intensity of transmission, as opposed to larger adults that may feed less frequently but are also longer-lived, thereby increasing their transmission potential. [36, 41–44]-44]

It is worth noting that the increased tolerance to insecticide following metal pollutant exposure in both insecticide resistant and susceptible strains mirrors findings from Kenya where the presence of lead correlated positively with the presence of *An*. *gambiae* and *Ae*. *aegypti* larvae [10]. The high lethal doses tolerated by *An*. *arabiensis*, as well as the fact that lead does not affect larval development while augmenting insecticide tolerance, is an important consideration because lead exposure generally affects crucial life history traits in exposed mosquitoes.

When examining the biochemical basis of these responses, it appears that the key detoxification enzymes are the cytochrome P450s and β-esterases. No significant changes were noted in the α-esterases following exposure to metal pollutants and the only significant change in GST activity was decreased activity in insecticide resistant females. Beta esterases were not affected in the insecticide susceptible strain, but increases were noted in males and females of the resistant strain. For the cytochrome P450s, cadmium exposure induced an increase in the activity of insecticide susceptible females. For insecticide resistant mosquitoes, two of three treatments increased cytochrome P450 activity in both males and females. Cytochrome P450 activity is crucial in metal tolerance. Metal tolerant *An*. *gambiae* females generally showed increased CYP6 expression, while males typically showed decreased CYP6M2, CYP6P3 and CYP6Z1 expression [23]. It is important to note that the data from this study was obtained from metal naïve populations as opposed to metal tolerant strains, in contrast to the Musasia *et al*. [23] study. This study suggests that an interplay between the Phase I enzymes the β-esterases and the cytochrome P450 proteins may be the key mediators in increased insecticide tolerance in adults eclosed from larvae exposed to metal pollutants.

Oxidative stress defence plays an important role in insecticide resistance [30, 45] with increased oxidative stress defence playing a key role in insecticide tolerance [33]. Understanding the effect that larval metal pollution has on adult oxidative stress response is therefore important, not only because of the potential effect on the expression of insecticide resistance in adult mosquitoes, but also because metal pollution is also a major source of oxidative stress in numerous organisms [46]. Glutathione peroxidase activity displayed a sex-specific induction in adults after larval metal exposure. While pollutant exposure reduced peroxidase activity in susceptible males, it increased the activity in females. Similarly, in resistant females, activity was significantly increased, while no changes were noted in males. Superoxide dismutase activity was increased in all treatments except cadmium treatment in susceptible females. Increased defence against oxidative stress following exposure to metal pollutants likely advantages adult mosquitoes in an insecticide polluted environment because defence against oxidative damage also inadvertently leads to enhanced expression of certain insecticide resistance phenotypes via increased expression of GSTs [33].

It is concluded from this study that there is an enzyme-mediated positive link between tolerance to metal pollutants and insecticide resistance in adult *An*. *arabiensis* mosquitoes. Furthermore, exposure of larvae to metal pollutants may have operational consequences under an insecticide-based vector control scenario by increasing the expression of insecticide resistance in adults, and by affecting certain life history traits. Of the three metals examined, copper nitrate was the most toxic, while neither insecticide resistant nor susceptible larvae were unduly affected by exposure to lead.

## Supporting information

S1 Table Concentrations used to determine lethal concentrations of metals salts. The exact concentration points used to determine the LD50 values for each strain are detailed in this table.
(XLSX)

S2 Table Supplementary developmental parameters. The days of first and final pupation, as well as final pupation percentages for both SENN and SENN after metal treatment are detailed in this table.
(XLSX)

## References

[1] Zhang L, Liao Q, Shao S, Zhang N, Shen Q, Liu C. Heavy Metal Pollution, Fractionation, and Potential Ecological Risks in Sediments from Lake Chaohu (Eastern China) and the Surrounding Rivers. Int J Environ Res Public Health. 2015;12(11):14115–31. Epub 2015/11/13. doi: 10.3390/ijerph121114115 26561822 10.3390/ijerph121114115 PMC4661636

[2] Nkya TE, Akhouayri I, Kisinza W, David JP. Impact of environment on mosquito response to pyrethroid insecticides: facts, evidences and prospects. Insect Biochem Mol Biol. 2013;43(4):407–16. Epub 2012/11/06. doi: 10.1016/j.ibmb.2012.10.006 23123179 10.1016/j.ibmb.2012.10.006

[3] Walters CR, Somerset VS, Leaner JJ, Nel JM. A review of mercury pollution in South Africa: current status. J Environ Sci Health A Tox Hazard Subst Environ Eng. 2011;46(10):1129–37. Epub 2011/08/03. doi: 10.1080/10934529.2011.590729 21806457 10.1080/10934529.2011.590729

[4] Eisler R. Mercury hazards from gold mining to humans, plants, and animals. Rev Environ Contam Toxicol. 2004;181:139–98. Epub 2004/01/24. 14738199 10.1007/0-387-21733-9_4

[5] Huber M, Welker A, Helmreich B. Critical review of heavy metal pollution of traffic area runoff: Occurrence, influencing factors, and partitioning. Sci Total Environ. 2016;541:895–919. Epub 2015/10/09. doi: 10.1016/j.scitotenv.2015.09.033 26448594 10.1016/j.scitotenv.2015.09.033

[6] Jiao W, Chen W, Chang AC, Page AL. Environmental risks of trace elements associated with long-term phosphate fertilizers applications: a review. Environ Pollut. 2012;168:44–53. Epub 2012/05/18. doi: 10.1016/j.envpol.2012.03.052 22591788 10.1016/j.envpol.2012.03.052

[7] Sinka M, Bangs M, Manguin S, Coetzee M, Mbogo C, Hemingway J, et al The dominant *Anopheles* vectors of human malaria in Africa, Europe and the Middle East: occurrence data, distribution maps and bionomic precis. Parasit Vectors. 2010;3(1):117. 21129198 10.1186/1756-3305-3-117 PMC3016360

[8] Awolola TS, Oduola AO, Obansa JB, Chukwurar NJ, Unyimadu JP. Anopheles gambiae s.s. breeding in polluted water bodies in urban Lagos, southwestern Nigeria. J Vector Borne Dis. 2007;44(4):241–4. Epub 2007/12/21. 18092529

[9] Djouaka RF, Bakare AA, Bankole HS, Doannio JM, Kossou H, Akogbeto MC. Quantification of the efficiency of treatment of *Anopheles gambiae* breeding sites with petroleum products by local communities in areas of insecticide resistance in the Republic of Benin. Malar J. 2007;6:56 P doi: 10.1186/1475-2875-6-56 17488523 10.1186/1475-2875-6-56 PMC1885267

[10] Mireji PO, Keating J, Kenya E, Mbogo C, Nyambaka H, Osir E, et al Differential Induction of Proteins in *Anopheles gambiae* sensu stricto (Diptera: Cullicidae) Larvae in Response to Heavy Metal Selection. Int J Trop Insect Sci. 2006;26(4):214–26. Epub 2006/12/01. doi: 10.1017/S1742758406658955 20651951 10.1017/S1742758406658955 PMC2908035

[11] Antonio-Nkondjio C, Fossog BT, Ndo C, Djantio BM, Togouet SZ, Awono-Ambene P, et al *Anopheles gambiae* distribution and insecticide resistance in the cities of Douala and Yaounde (Cameroon): influence of urban agriculture and pollution. Malar J. 2011;10:154 Epub 2011/06/10. doi: 10.1186/1475-2875-10-154 ; 21651761 10.1186/1475-2875-10-154 PMC3118161

[12] Jones CM, Toe HK, Sanou A, Namountougou M, Hughes A, Diabate A, et al Additional selection for insecticide resistance in urban malaria vectors: DDT resistance in Anopheles arabiensis from Bobo-Dioulasso, Burkina Faso. PLoS One. 2012;7(9):e45995 Epub 2012/10/11. doi: 10.1371/journal.pone.0045995 23049917 10.1371/journal.pone.0045995 PMC3457957

[13] Poupardin R, Reynaud S, Strode C, Ranson H, Vontas J, David JP. Cross-induction of detoxification genes by environmental xenobiotics and insecticides in the mosquito Aedes aegypti: impact on larval tolerance to chemical insecticides. Insect Biochem Mol Biol. 2008;38(5):540–51. Epub 2008/04/15. doi: 10.1016/j.ibmb.2008.01.004 18405832 10.1016/j.ibmb.2008.01.004

[14] Poupardin R, Riaz MA, Jones CM, Chandor-Proust A, Reynaud S, David JP. Do pollutants affect insecticide-driven gene selection in mosquitoes? Experimental evidence from transcriptomics. Aquat Toxicol. 2012;114–115:49–57. Epub 2012/03/13. doi: 10.1016/j.aquatox.2012.02.001 22406618 10.1016/j.aquatox.2012.02.001

[15] Riaz MA, Poupardin R, Reynaud S, Strode C, Ranson H, David JP. Impact of glyphosate and benzo[a]pyrene on the tolerance of mosquito larvae to chemical insecticides. Role of detoxification genes in response to xenobiotics. Aquat Toxicol. 2009;93(1):61–9. Epub 2009/05/08. doi: 10.1016/j.aquatox.2009.03.005 19419775 10.1016/j.aquatox.2009.03.005

[16] Nkya TE, Poupardin R, Laporte F, Akhouayri I, Mosha F, Magesa S, et al Impact of agriculture on the selection of insecticide resistance in the malaria vector *Anopheles gambiae*: a multigenerational study in controlled conditions. Parasit Vectors. 2014;7:480 Epub 2014/10/17. doi: 10.1186/s13071-014-0480-z 25318645 10.1186/s13071-014-0480-z PMC4201709

[17] Rayms-Keller A, Olson KE, McGaw M, Oray C, Carlson JO, Beaty BJ. Effect of heavy metals on *Aedes aegypti* (Diptera: Culicidae) larvae. Ecotoxicol Environ Saf. 1998;39(1):41–7. doi: 10.1006/eesa.1997.1605 9515074 10.1006/eesa.1997.1605

[18] Mireji PO, Keating J, Hassanali A, Mbogo CM, Muturi MN, Githure JI, et al Biological cost of tolerance to heavy metals in the mosquito *Anopheles gambiae*. Med Vet Entomol. 2010;24(2):101–7. Epub 2010/04/09. doi: 10.1111/j.1365-2915.2010.00863.x 20374478 10.1111/j.1365-2915.2010.00863.x PMC2921613

[19] Chin TA, Templeton DM. Protective elevations of glutathione and metallothionein in cadmium-exposed mesangial cells. Toxicology. 1993;77(1–2):145–56. Epub 1993/01/29. 8442010 10.1016/0300-483x(93)90145-i

[20] Coogan TP, Bare RM, Bjornson EJ, Waalkes MP. Enhanced metallothionein gene expression is associated with protection from cadmium-induced genotoxicity in cultured rat liver cells. J Toxicol Environ Health. 1994;41(2):233–45. Epub 1994/02/01. doi: 10.1080/15287399409531839 8301701 10.1080/15287399409531839

[21] Mattingly KS, Beaty BJ, Mackie RS, McGaw M, Carlson JO, Rayms-Keller A. Molecular cloning and characterization of a metal responsive *Chironomus tentans* alpha-tubulin cDNA. Aquat Toxicol. 2001;54(3–4):249–60. Epub 2001/08/08. 11489310 10.1016/s0166-445x(00)00181-8

[22] Mireji PO, Keating J, Hassanali A, Impoinvil DE, Mbogo CM, Muturi MN, et al Expression of metallothionein and alpha-tubulin in heavy metal-tolerant *Anopheles gambiae* sensu stricto (Diptera: Culicidae). Ecotoxicol Environ Saf. 2010;73(1):46–50. Epub 2009/09/09. doi: 10.1016/j.ecoenv.2009.08.004 19735939 10.1016/j.ecoenv.2009.08.004 PMC2783303

[23] Musasia FK, Isaac AO, Masiga DK, Omedo IA, Mwakubambanya R, Ochieng R, et al Sex-specific induction of CYP6 cytochrome P450 genes in cadmium and lead tolerant *Anopheles gambiae*. Malar J. 2013;12:97 doi: 10.1186/1475-2875-12-97 23497268 10.1186/1475-2875-12-97 PMC3601984

[24] Dandalo LC. Brooke BD, Munhenga G, Lobb LN, Zikhali J, Ngxongo SP et al Population Dynamics and *Plasmodium falciparum* (Haemosporida: Plasmodiiae) infectivity rates for the Malaria vector *Anopheles arabiensis* (Diptera: Culicidae) at Mamfene, KwaZulu-Natal, South Africa. J Med Ento. 2017 11 7; 54 (6): 1758–1766 10.1093/jme/tjx169 28968846

[25] Kitau J, Oxborough RM, Tungu PK, Matowo J, Malima RC, Magesa SM, et al Species shifts in the *Anopheles gambiae* complex: do LLINs successfully control *Anopheles arabiensis*? PLoS One. 2012;7(3):e31481 doi: 10.1371/journal.pone.0031481 22438864 10.1371/journal.pone.0031481 PMC3306310

[26] Sharp BL, Le Sueur D, B P. Effect of DDT on survival and blood feeding success of *Anopheles arabiensis* in northern Kwazulu, Republic of South Africa. J Am Mosq Control Assoc. 1990;6(2):197–202. 2370526

[27] Killeen GF. Characterizing, controlling and eliminating residual malaria transmission. Malar J. 2014;13:330 Epub 2014/08/26. doi: 10.1186/1475-2875-13-330 25149656 10.1186/1475-2875-13-330 PMC4159526

[28] Hunt RH, Brooke BD, Pillay C, Koekemoer LL, Coetzee M. Laboratory selection for and characteristics of pyrethroid resistance in the malaria vector *Anopheles funestus*. Med Vet Entomol 2005;19(3):271–5. doi: 10.1111/j.1365-2915.2005.00574.x 16134975 10.1111/j.1365-2915.2005.00574.x

[29] Oliver SV, Brooke BD. The effect of elevated temperatures on the life history and insecticide resistance phenotype of the major malaria vector *Anopheles arabiensis* (Diptera: Culicidae). Malar J. 2017;16(1):73 Epub 2017/02/15. doi: 10.1186/s12936-017-1720-4 28193292 10.1186/s12936-017-1720-4 PMC5307775

[30] Oliver SV, Brooke BD. The effect of multiple blood-feeding on the longevity and insecticide resistant phenotype in the major malaria vector *Anopheles arabiensis* (Diptera: Culicidae). Parasit Vectors. 2014;7:390 Epub 2014/08/26. doi: 10.1186/1756-3305-7-390 25150975 10.1186/1756-3305-7-390 PMC4161849

[31] Oliver SV, Brooke BD. The effect of larval nutritional deprivation on the life history and DDT resistance phenotype in laboratory strains of the malaria vector *Anopheles arabiensis*. Malar J. 2013;12:44 Epub 2013/02/02. doi: 10.1186/1475-2875-12-44 23368928 10.1186/1475-2875-12-44 PMC3570311

[32] Matambo TS, Abdalla H, Brooke BD, Koekemoer LL, Mnzava A, Hunt RH, et al Insecticide resistance in the malarial mosquito *Anopheles arabiensis* and association with the *kdr* mutation. Med Vet Entomol. 2007;21(1):97–102. Epub 2007/03/22. doi: 10.1111/j.1365-2915.2007.00671.x 17373952 10.1111/j.1365-2915.2007.00671.x

[33] Oliver SV, Brooke BD. The Role of Oxidative Stress in the Longevity and Insecticide Resistance Phenotype of the Major Malaria Vectors *Anopheles arabiensis* and *Anopheles funestus*. PLoS One. 2016;11(3):e0151049 Epub 2016/03/11. doi: 10.1371/journal.pone.0151049 26964046 10.1371/journal.pone.0151049 PMC4786153

[34] WHO. Test procedures for insecticide resistance monitoring in malaria vector mosquitoes. 2016. http://wwwwhoint/iris/handle/10665/250677.

[35] Finney DJ. Probit Analysis. 2nd ed: Cambridge University Press, New York; 1952.

[36] Lyimo EO, Koella JC. Relationship between body size of adult *Anopheles gambiae s*.*l*. and infection with the malaria parasite *Plasmodium falciparum*. Parasitology. 1992;104 (Pt 2):233–7. Epub 1992/04/01. 1594289 10.1017/s0031182000061667

[37] Oliver SV, Brooke BD. The effects of ingestion of hormonal host factors on the longevity and insecticide resistance phenotype of the major malaria vector *Anopheles arabiensis* (Diptera: Culicidae). PLoS One. 2017 7 11;12(7):e0180909 doi: 10.1371/journal.pone.0180909 eCollection 2017 28700639 10.1371/journal.pone.0180909 PMC5507448

[38] Chambers GM, Klowden MJ. Age of *Anopheles gambiae* Giles male mosquitoes at time of mating influences female oviposition. J Vector Ecol. 2001;26(2):196–201. Epub 2002/01/30. 11813657

[39] Sawadogo SP, Diabate A, Toe HK, Sanon A, Lefevre T, Baldet T, et al Effects of age and size on *Anopheles gambia*e s.s. male mosquito mating success. J Med Entomol. 2013;50(2):285–93. Epub 2013/04/02. 23540115 10.1603/me12041

[40] Padmanabha H, Lord CC, Lounibos LP. Temperature induces trade-offs between development and starvation resistance in *Aedes aegypti* (L.) larvae. Med Vet Entomol. 2011;25(4):445–53. Epub 2011/03/18. doi: 10.1111/j.1365-2915.2011.00950.x 21410734 10.1111/j.1365-2915.2011.00950.x PMC3136550

[41] Suwanchaichinda C, Paskewitz SM. Effects of larval nutrition, adult body size, and adult temperature on the ability of *Anopheles gambiae* (Diptera: Culicidae) to melanize sephadex beads. J Med Entomol. 1998;35(2):157–61. Epub 1998/04/16. 9538577 10.1093/jmedent/35.2.157

[42] Lehmann T, Dalton R, Kim EH, Dahl E, Diabate A, Dabire R, et al Genetic contribution to variation in larval development time, adult size, and longevity of starved adults of *Anopheles gambiae*. Infect Genet Evol. 2006;6(5):410–6. Epub 2006/03/10. doi: 10.1016/j.meegid.2006.01.007 16524787 10.1016/j.meegid.2006.01.007

[43] Takken W, Smallegange RC, Vigneau AJ, Johnston V, Brown M, Mordue-Luntz AJ, et al Larval nutrition differentially affects adult fitness and *Plasmodium* development in the malaria vectors *Anopheles gambiae* and *Anopheles stephensi*. Parasit Vectors. 2013;6(1):345 Epub 2013/12/12. doi: 10.1186/1756-3305-6-345 24326030 10.1186/1756-3305-6-345 PMC4029273

[44] Shiao SH, Hansen IA, Zhu J, Sieglaff DH, Raikhel AS. Juvenile hormone connects larval nutrition with target of rapamycin signaling in the mosquito *Aedes aegypti*. J Insect Physiol. 2008;54(1):231–9. Epub 2007/11/06. doi: 10.1016/j.jinsphys.2007.09.007 17981294 10.1016/j.jinsphys.2007.09.007 PMC2242809

[45] Vontas JG, Small GJ, H. J. Glutathione S-transferases as antioxidant defence agents confer pyrethroid resistance in *Nilaparvata lugens*. Biochem J 2001;357(Pt 1):65–72. 11415437 10.1042/0264-6021:3570065 PMC1221929

[46] Monserrat JM, Martinez PE, Geracitano LA, Amado LL, Martins CM, Pinho GL, et al Pollution biomarkers in estuarine animals: critical review and new perspectives. Comp Biochem Physiol C Toxicol Pharmacol. 2007;146(1–2):221–34. Epub 2006/10/19. doi: 10.1016/j.cbpc.2006.08.012 17045848 10.1016/j.cbpc.2006.08.012

